# Haploid Embryogenesis in Isolated Microspore Culture of Carrots (*Daucus carota* L.)

**DOI:** 10.3390/life11010020

**Published:** 2020-12-31

**Authors:** Natalia Shmykova, Elena Domblides, Tatiana Vjurtts, Arthur Domblides

**Affiliations:** Federal State Budgetary Scientific Institution Federal Scientific Vegetable Center (FSBSI FSVC), VNIISSOK, 143072 Moscow Region, Russia; shmykovanat@mail.ru (N.S.); tajtzha@yandex.ru (T.V.); arthurdom@inbox.ru (A.D.)

**Keywords:** DH plants-doubled haploids plants, *Daucus carota* L., culture of isolated microspores in vitro, embryogenesis, secondary embryoids, albino plants

## Abstract

The process of embryogenesis in isolated microspore culture was studied in eight carrot accessions of different origin. The ½NLN-13 medium supplemented with 0.2 mg/L 2,4D and 0.2mg/L kinetin was used to induce embryogenesis. The temperature treatment was performed at 5–6 °C for three days, followed by cultivation at 25 °C in darkness. As was shown, the first embryogenesis was only observed in microspores at the late vacuolated stage when the nucleus moved from the center to one pole following the long cell axis. Depending on the nucleus position, the microspore can divide into two equal or two different sized cells. Following divisions occurred either in one of these cells or in two. However, microspores that divided into two unequal cells were morphologically different form bi-cellular pollen grain. Embryogenic divisions in bi-cellular pollen grains were not observed. First divisions began by the third day of cultivation, and continued until the globular embryoid stage that was well-seen after the fourth week of cultivation. The already-formed embryoids can develop the secondary embryoids on their surface. Depending on the genotype, up to 1000 secondary embryoids can be produced from one embryoid in the liquid MSm medium supplemented with 0.1 mg/L of kinetin for regeneration. All carrot accessions studied were split into three groups: responsive genotypes, weakly responsive genotypes, and reluctant genotypes. The highest yield was 53 initial embryoids per a 6 cm diameter petri dish. Thus, the Nantskaya 4 cultivar totally produced 256 initial embryoids, out of which 94 developed into green plantlets and 162 into albino plantlets, whereas 97 initial embryoids with 45 albino plantlets formed from them were obtained from Chantenay cultivar.

## 1. Introduction

In world practice, despite the fact that carrots (*Daucus carota* L.) are an economically important vegetable crop, doubled haploid plants (DH-plants) in isolated microspore culture in vitro were first obtained only at the end of the first decade of the twenty-first century. The first studies began in the eighties in Denmark and Russia, then in the nineties in Japan, which showed the possibility of obtaining homozygous plants in the anther culture [1,2,3]. Further studies were continued in Russia, Poland, and China [4,5,6]. However, the yield of doubled haploid plants in the carrot anther culture was low. At the same time, the anther culture has a big drawback, since embryoids or callus can develop not only from microspores, but also from somatic tissues of the anther [7], which complicates their use in breeding. The second disadvantage of the anther culture is the laborious process of isolating the anthers from the buds, because their sizes are insignificant (1–1.5 mm). Microspore culture is simpler and more effective than anther culture. The feasibility of in vitro cultivation of microspores for carrots was demonstrated by Matsubara et al. (1995) [8]. However, tangible results in isolated microspore culture in vitro of carrots were obtained much later. In 2010, Gorecka et al. [9] obtained 42 doubled haploid plants in a carrot microspore culture in vitro. Ferrie et al. (2011) [10] created 17 homozygous carrot lines, and Li et al. (2013) [11] observed the formation of embryoids and callus in 28 cultivars. Stable embryoid yield in culture of isolated microspores in vitro and DH-plants were obtained from eight varieties of *D. carota* L. using five different nutrient media [12]. From the above material, it becomes clear that studies on embryogenesis in the culture of carrot microspores are few in comparison with crops such as tobacco, rapeseed, wheat, barley, and rice, for which it has been shown that a large number of factors affect the efficiency of pollen embryogenesis, including the culture conditions and genotypes of donor plants [13,14]. However, among these factors, the most important are the stages of development of microspores and pollen grains, which determine success in the induction of embryogenesis. In most studies on other plant species, it has been shown that vacuolated microspores and an early two-celled pollen grain are the most optimal for switching from a gametophytic development path to a sporophytic one [15,16,17,18]. However, more precise characteristics of the stage of development of carrot microspores, at which the cell is able to change its program, still need to be determined. This will help to understand the process of transition from the gametophytic path of development to the sporophytic one, and to select a more efficient population of microspores for the induction of embryogenesis. Moreover, there is no detailed description of the embryogenesis process (the pathway of division of embryogenic microspores) in carrots.

The purpose of this study is to conduct a cytological study of the stages of development of carrot microspores and to reveal the morphological features of the optimal stage at which a transition from gametophytic to sporophytic development is possible. The purpose of this study was also to determine the peculiarities of embryogenesis in carrot genotypes with contrasting embryogenic ability, and to give a step-by-step description of the possible pathways for the development of microspores/pollen grains in in vitro culture.

## 2. Materials and Methods

### 2.1. Plant Material and Plants Growing Conditions

The eight accessions of carrot (*Daucus carota* L.) from the collection of the Federal State Budgetary Scientific Institution Federal Scientific Vegetable Center (FSBSI FSVC), the Federal State Budgetary Scientific Institution Federal Research Center, and the N.I.Vavilov All-Russian Institute of Plant Genetic Resources, VIR were used in the present study. Vernalized carrot roots were planted in plastic pots of 22 cm diameter filled with a mixture of peat and perlite (7:3, *v*/*v*). Planting was done three times, in January, February, and March. Pots were placed in a growth chamber with a 16 h photoperiod, a temperature of 19 °C, and an illuminance of 8000 lux (lamp: horturion HPS, 600 W, 220 V, E40). Donor plants were watered as needed and fertilized once a week with a liquid of commercial fertilizer, Akvarin, containing N (13%), P_2_O_5_ (5%), K_2_O (25%), MgO (2%), S (8%), Fe (EDTA) (0.054%), Zn (EDTA) (0.014%), Cu (EDTA) (0.01%), Mn (EDTA) (0.042%), Mo (0.004%), and B (0.02%).

### 2.2. Microspore Culture

To prepare the microspore culture, the outermost umbellets with buds containing the late vacuolated microspores and young bi-cellular pollen grains were used [4]. The buds of the two outer rows were separated with tweezers. The buds were sterilized for 20 min in 2.5% sodium hypochlorite with the addition of Tween-20 (Panreac, Barselona, Spain) (one drop per 100 ml of solution), then rinsed with sterile distilled water three times for 10 min. Sterile buds were transferred to a 20 ml sterile bottle containing 6 ml of ½ NLN [19] medium supplemented with 13% (*w/v*) sucrose (designated as ½ NLN-13) and macerated by a sterile magnetic stirrer on the magnetic stirring bar. The crude suspension was filtered through a 40 µm nylon screen into a 50 ml centrifuge tube, and then centrifuged at 120 g for 5 min. Supernatant was decanted and 15–20 ml of ½ NLN-13 was added to re-suspend the pellet. This procedure was repeated twice for a total of three washes. The rinsed microspores were suspended in ½ NLN-13 medium supplemented with 0.2 mg/L of 2,4-Dichlorophenoxyacetic acid (2,4-D) and 0.2 mg/L of 6-furfurylaminopurine (kinetin). The density of microspores was adjusted to 1 × 10^5^ microspores/ml in the same medium. The microspore suspension was dispensed at 5 mL per petri dish (60 mm × 1.5 mm). The petri dishes were incubated in the dark at 5–6 °C for three days for cold pretreatment, and then maintained at 25 °C under dark conditions to induce embryogenesis during 4–5 weeks.

### 2.3. Plant Regeneration

The embryos in the globular stage were transferred to an MSm [20] solid medium with 2% sucrose, 0.1 mg/L kinetin, and 0.7% agar, and were cultured at 22 °C during a 16 h photoperiod at an illuminance of 3000 Lx for 6–7 weeks. Developed embryos and/or embryogenic calli were transferred to filter paper bridges in glass tubes (20 mm × 200 mm) with a liquid MSm medium of the same content and cultured at the same conditions for 7–8 weeks.

The six-well culture plate (Corning), containing 1 ml/well of liquid MSm medium with 2% sucrose and 0.1 mg/L of kinetin, was used for the study of secondary embryogenesis. Each embryoid was placed in the well and was cultured for four weeks on an orbital shaker (50 rpm, gentle shaking) at 22 °C during a 16 h photoperiod at an illuminance of 3000 Lx.

SIGMA reagents marked “plant cell culture tested” were used for the experiments.

### 2.4. Plantlets Growing

The plantlets with healthy roots and leaves were transplanted into vegetation vessels with a mixture of peat and perlite (7:3, *v*/*v*), and were covered by perforated plastic cups for acclimatization to the in vivo conditions. Regenerated plants were grown in the same conditions as the donor plants.

### 2.5. Cytological Analysis 

To study the stages of microspore development, the buds were fixed in a mixture of 96% ethanol and glacial acetic acid (3:1, *v*/*v*), and then rinsed three times with 70% ethanol. The fixed materials were stained in 2.0% acetocarmine by keeping them for three or four days at room temperature, or for any longer period in a refrigerator. Microspores isolated from anthers were studied using a light microscope Axio Imager.A2 with the camera AxioCam MRc5 (Carl Zeiss Microscopy GmbH, Jena, Germany) and with the AxioVision Rel 4.8 program. Study of embryogenesis in the isolated microspore culture was conducted every 3–7 days during the 1.5–2 months using an inverted microscope Primo Vert (Carl Zeiss Microscopy GmbH, Jena, Germany) with the camera AxioCam ERc5s and the stereo microscope Stemi 508 with the camera Axiocam 305 color (Carl Zeiss Microscopy GmbH, Jena, Germany). Three experiments (according to the time of planting of carrot donor plants) were conducted with five replicates.

### 2.6. Ploidy Level Determination

The ploidy of regenerated plants was determined by flow cytometry of cell nuclei. It was performed on the basis of the bioengineering laboratory of Altai State University in Barnaul using a Partec CyFlow PA flow cytometer (Partec GmbH, Münster, Germany) with a laser radiation source and a wavelength of 532 nm. Approximately 0.5–1.0 cm^2^ of plant tissue was cut with a razor blade in the presence of 1 ml of lysis buffer: 0.2 M of Tris, 4 mM of MgCl_2_, 50 µg/mL of RNA-ase, 0.5% (*v*/*v*) TRITON X-100, 0.5% (*v*/*w*) polyvinylpyrolidone K15, and 50 µg/mL of propidium iodide, with a pH of 7.5 (Skaptsov et al.,2016) [21]. 

The diploid (2n = 2x = 18) plants of carrots were used as external standards to determine the ploidy. Visualization and plot constriction were performed using Flowing Software 2.5.1. (University of Turku, Turku, Finland). Data observed were calculated with XLStat software (https://www.xlstat.com/en/) (Addinsoft).

### 2.7. Statistical Analysis

For each genotype, isolated microspores in at least five petri dishes were cultivated. The experiment was repeated at least three times. Statistical analysis was performed using one-way analysis of variance (ANOVA), and means were compared using Student’s *t*-test with a probability of 99%. The statistical analyses were carried out using Microsoft Excel 2010 for Windows 10.

## 3. Results

### 3.1. Study of the Development of Carrot Microspores

It is extremely important to correctly determine the phase of development of microspores in buds for embryogenesis in a culture of isolated microspores in vitro. The used method of staining anthers with acetocarmine made it possible to clearly determine the location of the nucleus and vacuole and the state of the cell wall at each stage of microsporogenesis.

**Figure 1:**
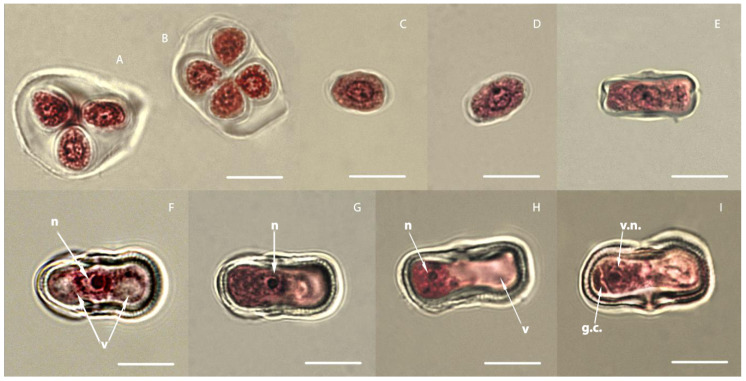
Developmental stages of carrot microspores and pollen. (**A**) Tetrahedral arrangement of microspores in a tetrad; (**B**) tetragonal location of microspores in the tetrad; (**C**) microspore of a round shape with a thin shell, just released from the tetrad (stage microspore 1); (**D**) microspore of round-elliptical shape with a thin shell (stage microspore 1); (**E**) an elliptical microspore with a visible envelope, a centrally located nucleus, and small vacuoles on both sides of the nucleus (stage microspore 2); (**F**) an elliptical microspore with a centrally located nucleus and two large vacuoles on either side of the nucleus; transverse thickenings/ribs begin to appear in the thickening shell (stage microspore 3); (**G**) microspore with a centrally located nucleus and large forming vacuoles on one side of the nucleus (stage microspore 4: beginning); (**H**) microspore with a nucleus located at one of the poles and a large vacuole that occupies most of the microspore (stage microspore 4); (**I**) two-celled pollen grain. n—nucleus; v—vacuole; vn—vegetative nucleus; gc—generative cell. Bars = 10 μm.

Microsporogenesis in carrots begins with meiosis, which occurs in the mother cell of the microspore and leads to the formation of tetrads; each cell already contains a haploid set of chromosomes. Meiosis in the anther of carrots occurs asynchronously. Microspore tetrads are formed in a simultaneous manner. Microspores in tetrads are located tetrahedrally (Figure 1A) and, less often, tetragonally (Figure 1B). Both of these types can be found in the same pollen sac. After tetrads fall apart, the sequential development of the microspore begins. We have identified four stages in the development of carrot microspores [22], which are easily identified by the position of the nucleus and vacuoles and the state of the cell wall (Figure 1). The first stage of microspore development lasts from the moment the microspore is released from the callose wall of the tetrad until it acquires an elliptical shape. It is characterized by a large centrally located nucleus and dense cytoplasm. The cell wall of the microspore at this stage is thin, and vacuoles in the cytoplasm are small and barely noticeable (Figure 1C,D). The microspore volume increases with growth, they become elliptic in shape, and slightly noticeable vacuoles appear in them. During the second stage, the microspore is characterized by a thicker cell wall, a centrally located nucleus, and the formation of many small vacuoles on both sides of the nucleus. The shape of the microspore becomes more elongated, and its length increases (Figure 1E). The third stage is characterized by the presence of two large vacuoles on either side of the centrally located nucleus; in the thickening cell wall, a striation of the exine appears in cross-section (Figure 1F). The fourth stage begins with the displacement of the nucleus from the center to one of the poles, and as the nucleus moves forward, the vacuoles merge and form one large vacuole located at the opposite pole (Figure 1G,H). After the displacement of the nucleus, the microspore is gradually enriched in cytoplasm, and the vacuole begins to significantly decrease in size. After this stage, differentiating mitosis occurs, and because of this, a pollen grain is formed with a very large vegetative cell and a small generative lenticular cell (Figure 1I). If differentiating division is impaired, vacuoles are not formed and polarization of the cytoplasm does not occur. This will produce an abnormal pollen grain with equal or nearly equal cells. In both cases, the cells contain identical nuclei and do not have vacuoles. The resulting cells are difficult to define as generative or vegetative, since they differ only in size. In morphology, they resemble cells of the meristem tissue, i.e., we observe abnormal differentiation of pollen grain cells to varying degrees. Studies conducted earlier in our laboratory have shown that disruption of differentiating mitosis in carrots can occur not only in culture in vitro, but also in vivo. At the same time, the percentage of such abnormal pollen grains can reach up to 8.7%, and will depend on the genotype of the donor plant, the conditions of its cultivation, and the stress applied [22]. In our experiment, in three of the eight studied genotypes, immediately after the isolation of microspores and even before cultivation, microspores were present in the suspension, which were divided into two equal cells. The percentage of such abnormal microspores ranged from 2.0 to 5.7%, depending on the genotype (Table 1).

Usually, in the cell suspension immediately after introduction into the culture, carrot microspores can be at different stages of development, with one being predominant. Since receiving a suspension of microspores, all of the buds located in the two extreme rows of the umbellets are subjected to grinding.

### 3.2. Embryogenesis of Carrots In Vitro Depending on the Stage of Development of Microspores

Morphological changes occurring with microspores were observed after three days of cultivation and continued up to 40 days.

**Figure 2:**
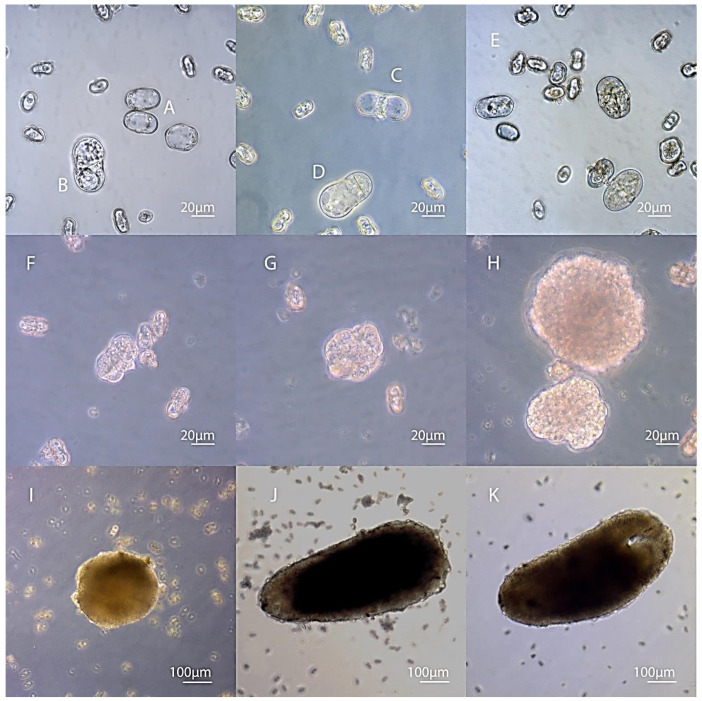
Microspore embryogenesis in *Daucus carota* L. (**A**) Microspores enlarged in size in three days of cultivation; (**B**) first microspore division (three days); (**C**) equal type of microspore division; (**D**) microspore divided into two cells unequal in volume; (**E**) in vitro culture at the proembryo formation stage (seven days); (**F**) proembryos formed by several cells and still surrounded by the exine (the microspore wall) on day 10 of cultivation; (**G**) release of the embryo-like structure from the original shell (14 days); (**H**) globular embryos (18 days); (**I**) globular embryos (24 days); (**J**) torpedo embryo (30 days); (**K**) cotyledonary embryos (35 days).

Microspores that were in the early stages of development (stages 1 and 2), when introduced into the culture, had a compressed cytoplasm by the third day of cultivation that had come off the walls of the microspore. Plasmolysis occurred in them. Apparently, the osmotic pressure in the nutrient medium was high for them. Microspores at a late, vacuolated stage (stage 4) were greatly enlarged by the third day, had a cytoplasm and a nucleus pressed against the walls, and the central part of the cell was occupied by a large vacuole (Figure 2A).

**Figure 3:**
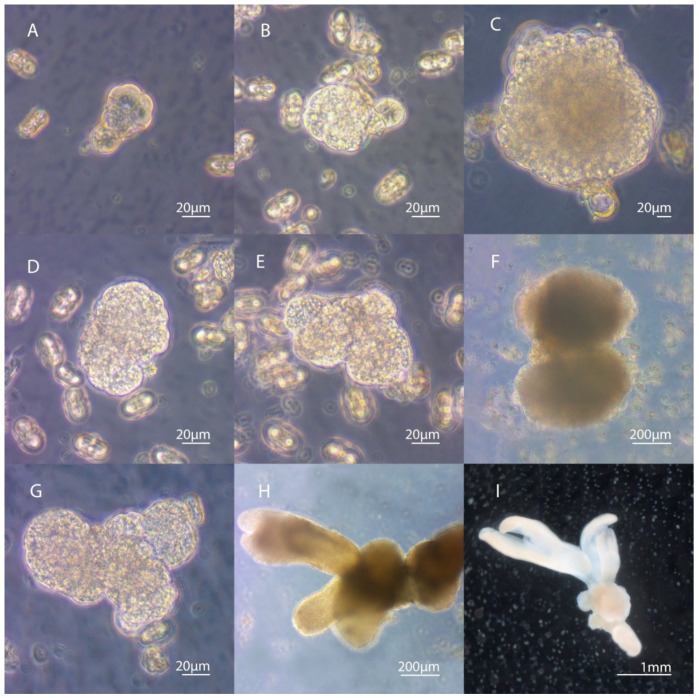
Different types of embryoid formation in microspore culture. (**A**–**C**) The formation of an embryoid from only one cell after equal division of the microspore. The second cell does not develop and remains in the form of a suspensor-like structure. (**D**–**F**) The development of twin embryos connected to each other. Embryoids are formed by the simultaneous division in the two original cells. (**G**–**I**) Formation of interconnected polyembryoids. (**I**) The polyembryo observed under the stereomicroscope.

Young two-celled pollen grains did not undergo visible morphological changes, and remained in this state for up to 20–30 days and gradually degraded. It is noteworthy that under in vivo conditions, during development inside the anther, this stage takes an insignificant period of time (less than a day), and then, after the second mitosis, a three-celled pollen grain is formed. Microspores at a stage optimal for transition to the sporophytic path of development (with a nucleus located at one of the poles and a large vacuole that occupies most of the microspore) underwent a number of changes in in vitro culture. The first divisions of cells increased in size were observed after 3–5 days of cultivation (Figure 2B). By the end of the third week, the initially enlarged cells either divided and formed multicellular formations or plasmolysis occurred in them, the vacuole disappeared, and the compressed cytoplasm was located in the center of the microspore. In the initial microspore at stage 4, two types of division were observed: the first was division into two equal cells (Figure 2C), and the second was when one cell was large and the other cell was small. However, this two-celled structure formation did not look like a two-celled pollen grain, and although a smaller cell was in the place of a generative cell, it was morphologically very different from it (Figure 2D). During this period, further divisions of both or one of the cells occurred in separate two-cell formations (Figure 2 and Figure 3). 

Initial cell divisions occur within the pollen grain shell, which can persist for a sufficiently long time, undergoing stretching. After 14–20 days of cultivation with cold pre-treatment in the culture of carrot microspores, multicellular formations appeared within the shell of the pollen grain (Figure 2F). In the responsive varieties Nantskaya 4 and Chantenay, they rapidly increased in size, while in the weakly responsive varieties, they remained small. 

We have noted the formation of various types of embryoids in the culture of carrot microspores. If divisions inside a cell initially divided into two equal microspores occurred synchronously and symmetrically, most often this led to the formation of a single regular embryoid, reaching the globular stage of development by day 18–24 of cultivation. Sometimes, the development of the embryoid proceeded from only one cell (initially divided into two equal/unequal cells of the microspore), division in the second cell stopped, and the cell was present in the developing embryo in the form of a suspensor-like structure (Figure 3A–C). In the culture of carrot microspores, we observed the development of twin embryos (Figure 3D–F) and polyembryoid formation (Figure 3G–I).

**Figure 4:**
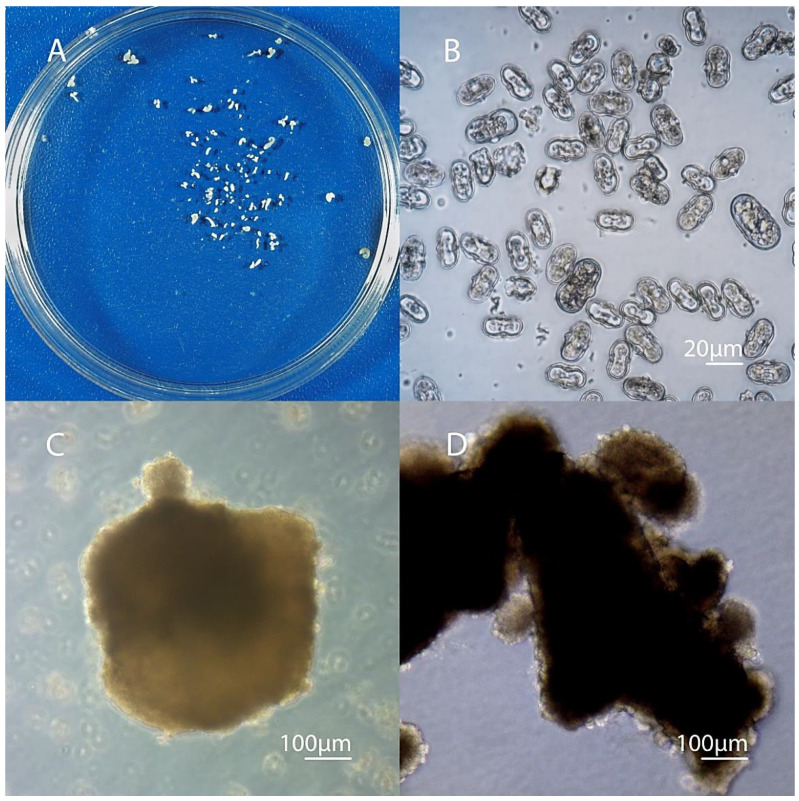
Morphological events in the culture of microspores on day 30–45 of cultivation. (**A**) Petri dish with the microspore-derived embryos produced in the culture of *Daucus carota* L. after 35 days of cultivation; (**B**) the death of proembryos/multicellular structures in weakly responsive genotypes after 30 days; (**C**) the formation of secondary embryoids on the surface of the globular embryo; (**D**) a large number of secondary embryoids on the surface of the cotyledon embryo (45 days).

After 30 days, globular embryoids could be seen with the naked eye in the culture of microspores of varieties Nantskaya 4 and Chantenay (Figure 2I). On day 35–40, part of the embryoids developed up to the cotyledon stage of development (Figure 2K, Figure 4A).

In weakly responsive cultivars, the death of multicellular structures began during this period (Figure 4B).

All studied varieties were divided into three groups. The first group included the responsive specimens Nantskaya 4 and Chantenay, from which doubled haploid plants were obtained. The highest yield was 53 initial embryoids per a 6 cm diameter petri dish. The second group consisted of weakly responsive varieties, in which only the formation of multicellular structures was observed without their further development. The third group included unresponsive ones, which had only single microspores with equal or unequal types of division into two cells, or there were no divisions (Table 1). ANOVA analysis showed that the share of influence of the genotype factor on the number of formed embryoids was 88%. Since it is known that a huge number of factors will influence the yield of doubled haploids, it is often necessary to optimize the technology for a specific genotype, choosing the optimal combination of factors (composition of the nutrient medium, temperature pretreatments, and cultivation conditions). The fact that multicellular structures were formed in weakly responsive varieties indicated that we were able to induce embryogenesis, but further cultivation conditions for these genotypes must be changed in order to obtain DH-plants.

### 3.3. Secondary Embryogenesis in the Culture of Carrot Microspores

**Figure 5:**
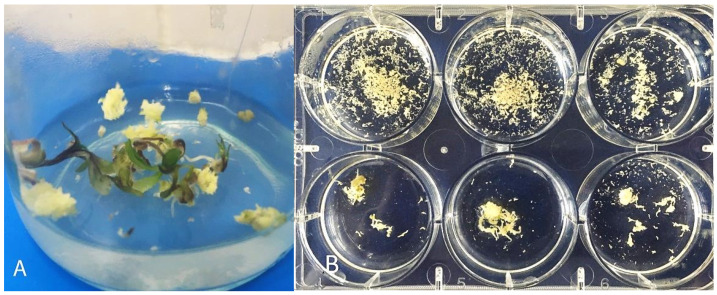
Formation of secondary carrot embryoids from one primary embryo after 21 days of cultivation. (**A**) On the solid MSm medium with 0.1mg/L of kinetin, 2% sucrose, and 0.7% agar; (**B**) on the liquid MSm medium with 0.1 mg/L of kinetin and 2% sucrose.

Carrots are a culture with a well-developed secondary embryogenesis. On an induction medium with 13% sucrose, the formation of secondary embryoids on the surface of primary embryoids was observed already from the globular stage (Figure 4C). After 40 days of cultivation at the cotyledon stage of development, the surfaces of the primary embryoids could be completely covered with numerous secondary embryoids (Figure 4D). Some of these embryoids could separate and develop independently, especially when using a shaker platform for cultivation. An experiment to study the process of secondary embryogenesis in carrots showed that one embryoid in a petri dish with a liquid medium for carrot regeneration (containing 2% sucrose and 0.1 mg/l of kinetin), after three weeks of cultivation and depending on the genotype, could form from 50 to 1000 secondary embryoids (Figure 5).

Responsive genotypes, after transferring embryos to a solid medium for regeneration from globular embryoids after three weeks, could often develop a callus with the formation of secondary embryoids, which often then formed the "polyembryoid" group. Both primary and secondary embryoids could grow directly into plants. The development of plants is especially good when the seedlings are transferred into test tubes with a liquid medium and bridges made of filter paper.

### 3.4. Development of Embryoids into Plant Regenerants Obtained in Culture of Carrot Microspores

**Figure 6:**
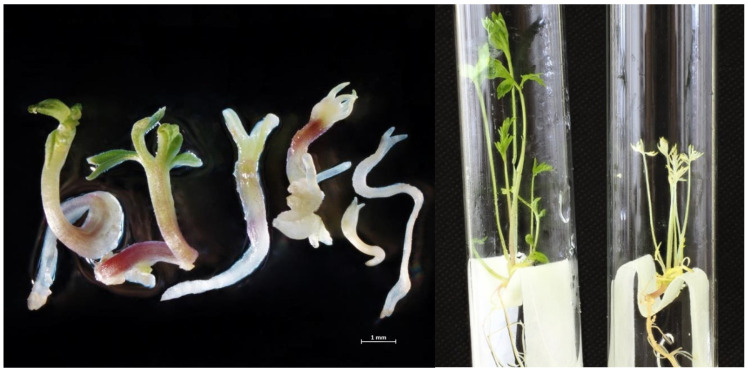
Green and albino plantlets developed from microspore-derived embryoids.

Some of the embryoids developed into albino plants, as they did not have green pigment. The number of albino plants depended on the genotype of the mother plant. Of the 256 embryoids of the carrot variety Nantskaya 4, 94 were green seedlings and 162 were albinos. Moreover, in the cultivar Chantenay, of 97 embryoids, 45 were albinos (Figure 6). Albino or partially albino embryoids developed very slowly, and the etiolated regenerant plants that formed from them were much smaller in size. Despite the formation of a sufficiently developed root system in them, it was not possible to adapt them to in vivo conditions, and therefore they all died.

Green seedlings in a liquid regeneration medium quickly formed a well-developed rosette of leaves and a root system (Figure 6). After 40–50 days, when the plant had 5–7 well-developed leaves and reached 10 cm in height, it could be transplanted into the soil for adaptation to in vivo conditions. Losses at the adaptation stage were about 20%. In total, we obtained 73 R_0_ regenerant plants from variety Nantskaya 4 and 39 from variety Chantenay. 

**Figure 7:**
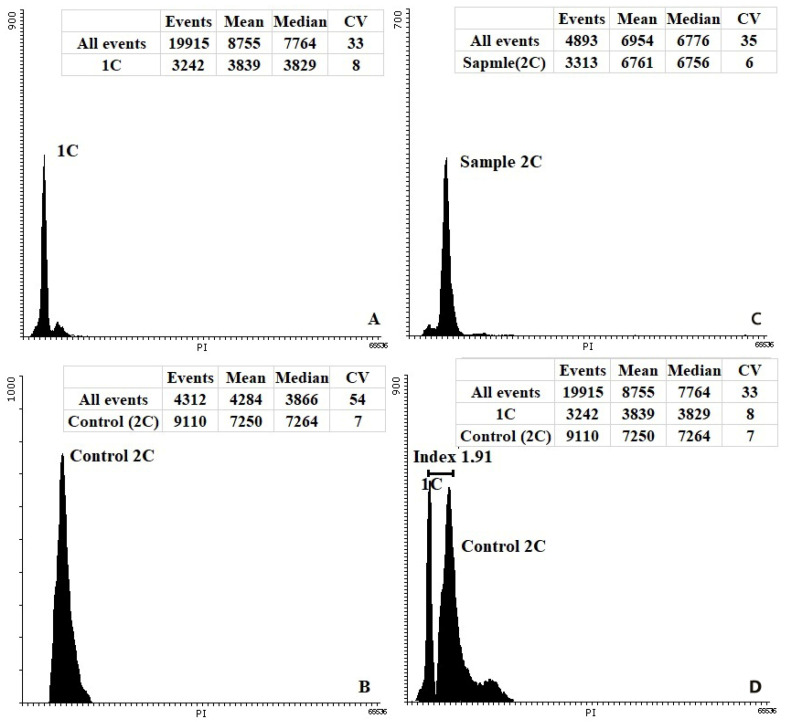
Flow cytometry histograms of carrot regenerants obtained from isolated microspore culture in vitro. External control samples were analyzed separately, without changing the cytometer settings. (**A**) Haploid; (**B**) the diploid (2n = 2x = 18) plants of carrots as a control; (**C**) diploid regenerated plant; (**D**) histogram of both the diploid control and haploid sample. Index: the difference between mean values of the peaks (mean).

**Figure 8:**
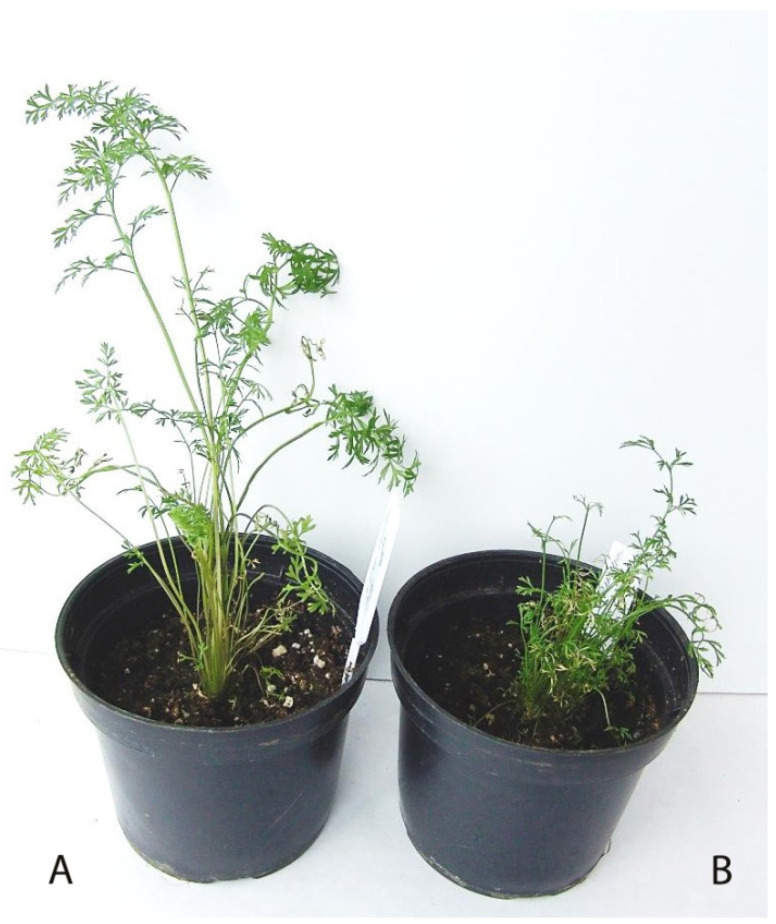
R_0_ regenerant plants from the Nantskaya 4 variety obtained from microspore-derived embryoids. (**A**) Doubled haploid plant with normal leaves; (**B**) haploid plant with short and thin dissected leaves.

In the study, plants regenerated from the primary individual embryos were established to test the ploidy level. Flow cytometry of leaf tissue (Figure 7) was mainly used for ploidy determination, along with additional cytological analysis (chromosome counting and chloroplast number in the stomata guard cells). In total, ploidy of 50 carrot regenerants of the Nantskaya 4 variety and 30 carrot regenerants of the Chantenay variety was examined (Table 2). Most of them (71.3 %) were diploids, 18.7% were haploids (Figure 8), and 10.0% were poliploids among the 80 plantlets tested.

## 4. Discussion

The process of induction of embryogenesis can be dependent or independent of exogenous growth regulators. There are effective microspore culture systems for plants such as tobacco, rapeseed, or pepper, whose induction is not influenced by hormones [23,24,25]. On the other hand, in the anther culture of wheat, triticale, and rye, a number of scientists use 2.4 D and kinetin in induction media [26,27,28,29,30,31]. Carrots are among those plants that require exogenous growth regulators for the induction of embryogenesis. Andersen et al. (1990) [32], Goreska et al. (2005) [5], Goreska et al. (2010) [9], Li et al. (2013) [11], and Kiszczak et al. [33] used 2.4 D in combination with NAA to induce embryogenesis. Hu et al. (1993) [3] and Matsubara et al. (1995) [8] for this purpose added 2.4 D and kinetin to the media, while Tyukavin et al. (1999) [4] added only 2.4 D. We obtained positive results when using 0.2 mg/L of 2.4 D and kinetin in the culture of microspores of carrot varieties Nantskaya 4 and Chantenay. Earlier, Shmykova and Tyukavin (2001) [22] showed that the pretreatment of inflorescences of carrot variety Nantskaya 4 with a solution containing 2 mg/l of 2.4 D leads to a violation of asymmetric mitosis of microspores in the anthers, i.e., in this case, 2.4 D can be considered as a trigger for the induction of embryogenesis in carrots.

The dynamics of embryogenic development of microspores strongly depend on the plant genotype and the cultivation method. In the work of Goreska et al. (2010) [9], cell structures visible with the naked eye were observed over a period of time from three weeks to six months. Li et al (2013) [11] noted the appearance of visible structures in some samples after five weeks of cultivation, while in others only after 4–6 months. The dynamics of the development of embryogenic structures in the culture of microspores of the carrot variety Nantskaya 4 and the culture of anthers of the same variety [4] practically coincided. The massive appearance of multicellular structures was observed by the end of the first month of cultivation in both experiments. The first divisions of microspores occurred after 3–5 days of cultivation using cold treatment for three days after introduction into the in vitro culture. It was the division of a greatly enlarged microspore containing a large central vacuole into two equal cells or one large and another small cell located in place of the generative one. Similar early changes have been demonstrated in rice microspore culture [34,35]. They showed that only large vacuolated rice microspores are capable of embryogenesis. The presence of large spherical microspores in the culture of carrot microspores was noted by Li et al. (2013) [11]. In our experiment, by the third and fourth weeks of cultivation, in some of these microspores, the formation of multicellular structures occurred from one or both cells of a two-celled pollen grain. This pathway of sporophytic development of carrot microspores is closest to the type B pathway [36], although it has individual characteristics. This refers to the division of the microspore into two cells that are not equal in size, but at the same time, the smaller cell is very different in its morphology compared to the generative one. This division can be explained by the cytological features of the carrot microspore, which at the stage of vacuolization is not round, but elliptical, and the movement of the nucleus before the first mitosis runs along the long axis. Apparently, disturbances in asymmetric mitosis occur in a time interval that occurs during the entire period of movement of the nucleus from the center to the periphery. Perhaps this is only one of the pathways of embryogenesis in carrots induced by 2.4 D, and it can be assumed that when other inducing factors are used, early two-cell pollen grains, which in this experiment remained unchanged, may also be involved in embryogenesis. Li et al. (2013) [11] identified two types of embryogenesis of microspores in carrots and attributed them to types C and E. The first type is callus formation: microspores initially increased to ellipses or spheres, followed by callus formation and development of embryoids from the callus. The second type of microspore development is direct embryogenesis, where the microspore was enlarged almost four times compared to its original size, and then an embryoid developed from it. In our experiment, the development of responsive specimens proceeded predominantly by direct embryogenesis, and callus formation was observed only in weakly responsive specimens. A characteristic feature for the culture of carrot microspores, which we would highlight, is a high ability for secondary embryogenesis. The formation of secondary embryoids on the surface of the primary embryoid begins already at the globular stage. At the same time, several secondary ones can form simultaneously on one embryoid, and they all begin to develop rather quickly forming embryoids of irregular shape, which can be mistaken for calluses. But unlike calluses, embryoids have a denser structure. This feature of carrots for the formation of secondary embryoids can lead to incorrect results when counting the number of embryos obtained in one petri dish. We recommend transferring the formed globular embryoids as early as possible onto a solid regeneration medium or into individual petri dishes with a liquid medium.

Temperature pretreatment with both elevated temperatures (32–35 °C) and low positive temperatures (2–6 °C) is the most common physiological effect used to induce embryogenesis of microspores. High efficiency of switching microspores from the gametophytic pathway to the sporophytic pathway was achieved with cold pretreatment on such crops as wheat [37], barley [38], rice [39], tobacco [40], and *Brassica rapa* [41]. High temperatures activated embryogenesis in such cultures as *Brassica napus* [42,43,44], *Capsicum annuum* [45], *Cucumis sativus* [46,47,48], and *Nicotiana tabacum* [23]. However, with regard to carrots, conflicting results have been obtained. Tyukavin (2007) [49] showed that the pretreatment of anthers in in vitro culture with both low and high temperatures, individually or in combination, is ineffective. At the same time, preliminary treatment of inflorescences with a low temperature of 4–6 °C for 48 hours doubled the number of anthers that formed embryoids. Li et al. (2013) [11] showed that the efficiency of temperature stress in carrot microspore culture depends on the genotype. In this experiment, DH carrot plants of varieties Nantskaya 4 and Chantenay were obtained by treating a culture of microspores at a temperature of 4–6 °C for three days. Apparently, low temperatures reduce cell degradation and reduce the breakdown of chemicals, reducing the toxicity of the environment.

The influence of genotype on embryogenesis has been shown in many well-studied crops, such as cereals [50] and plants of the *Brassica* genus [51,52,53]. Carrots are no exception. According to Andersen et al. (1990) [32], the yield of DH plants was 0.8% per 20,400 anthers. More successful results were obtained by Gorecka et al. (2005) [5], with 5.6 embryoids per 100 anthers. The efficiency of embryogenesis in this case depended on the variety sample: only six varieties out of 39 tested formed embryoids or calluses. Li et al (2013) [11] observed the formation of embryoids and calluses in 28 out of 47 cultivars. At the same time, they found a difference in the pathways of embryo development depending on the cultivar: 11 cultivars showed the C pathway, and 17 simultaneously had the C and E pathways. In our studies, out of eight cultivars, DH-plants were obtained from varieties Nantskaya 4 and Chantenay. However, the transition from the gametophytic pathway to the sporophytic pathway was noted for all samples. Half of them developed multicellular structures inside of the shell of the pollen grain, but compared to the Nantskaya 4 and Chantenay varieties, the growth of cells stopped inside this formation. Remarkable is the fact that these two genotypes had the highest percentage of formation of abnormally divided microspores (with an equal type of division) immediately after isolation: in Nantskaya 4, they were 8%, and in Chantenay, they were 5%. Apparently, the ability to disrupt differentiating mitosis is genetically inherent, and it is in these genotypes that it is possible to obtain the highest yield of doubled haploids in microspore culture in vitro using stress and plant growth regulators, such as 2.4 D. Obviously, in each specific case, it is necessary to develop individual protocols for the culture of isolated microspores for each specific plant species, variety, and genotype.

Albinism is considered to be the narrowest in breeding programs that use the anther or microspore method. Most of this applies to plants of the Poaceae family, in which the proportion of albino plants can range from 3% to 100% [54,55,56]. The influence of the donor plant genotype on the amount of albino plantlets formed during androgenesis was proved when screened in a full diallel population of four spelt wheat genotypes and 10 F1 hybrids [57]. The appearance of albino plants in dicotyledonous plants formed from microspores is a rather rare phenomenon. We are aware of the formation of albino seedlings obtained in an isolated culture of *Brassica purpuraria* microspores [58]. However, in our experiments, there were 63% albinos in Nantskaya 4 carrots. For plants of the family Poaceae, the formation of albino seedlings is primarily associated with the natural degradation of plastids in microspores of grain crops with the type of maternal inheritance [59]. In carrots, plastid inheritance occurs from both parents [60]. However, in both this and our earlier work, we obtained carrot seedlings obtained from microspores, which were devoid of completely green pigments [7,12]. Since according to Pacini et al. (1992) [61], regardless of the type of plastid inheritance, the degree of plastid differentiation may interfere with the entry of plastids into the generative cell. During the first haploid mitosis, plastids in microspores are in the form of proplastids or amyloplasts. If they enter the generative cell, then they are in the proplastid state. Apparently, disturbances in the separation of proplastids in carrots are associated with an unequal type of microspore division.

## 5. Conclusions

It was found that the induction of embryogenesis is possible only in carrot microspores, which are at a late stage of vacuolization during the movement of the nucleus from the center of the cell to the pole along the length of the cell axis. Depending on the position of the nucleus during the period of restructuring, the division of the microspore can occur both into two equal cells or cells differing in size. Subsequent divisions can occur in either one of these cells or both. However, the microspore divided into two unequal cells is morphologically very different from the two-celled pollen grain. No embryogenic divisions were observed in the two-celled pollen grain. High capacity for secondary embryogenesis is a characteristic feature of carrot microspore culture. The formation of secondary embryoids on the surface of the primary embryoid begins already from the globular stage, therefore, it is necessary to transfer the formed embryoids onto a solid nutrient medium or into separate petri dishes. Disturbances in the separation of proplastids in carrots associated with the unequal type of microspore division can lead to the formation of etiolated/albino seedlings, which, depending on the genotype, can account for up to 63% of the total number of regenerated seedlings.

## Figures and Tables

**Table 1:** Embryogenesis in isolated microspore culture of carrots (*Daucus carota* L.).

Accession	Abnormal Pollen Grains before Cultivation, (%)	The Number of Embryoids, Pcs/1 Petri Dish *		Embryoids Forming Green Seedlings/Albino Seedlings
		Maximum	Average	
Responsive Genotypes				
Nantskaya 4	5.7 ± 2.5	53	34.7 ± 11.3 ^a^	94/162
Chantenay	3.6 ± 1.5	37	25.2 ± 7.4 ^b^	52/45
Weakly Responsive Genotypes				
Scarlet Nantes (K-2030)	2 ± 1.0	5	2.5 ± 1.2 ^c^	0
Nantes Red ( Bp.k-2566)	0	3	1.3 ± 0.8 ^c^	0
Imperator (Bp.k.2569)	0	3	1.8 ± 0.9 ^c^	0
Reluctant Genotypes				
Naga (K-845)	0	0	0 ^d^	0
Tip Top (K-2332)	0	0	0 ^d^	0
Scarlet (Bp.k 2568)	0	0	0 ^d^	0
			LSD = 3.45	

Note: Values presented are means of three independent experiments with five replicates in each ± SE (standard error). Values marked with a similar letter had no significant differences at *p* ≤ 0.01. * Embryoids visible to the naked eye were counted in a petri dish (6 cm in diameter) at 35–40 days of cultivation.

**Table 2:** Ploidy level of carrot plants regenerated from microspore-derived embryoids.

Accession	No. of Plants	Ploidy Level					
		Haploid		Diploid		Polyploids	
		No.	%	No.	%	No.	%
Nantskaya 4	50	7	14.0	37	74.0	6	12.0
Chantenay	30	8	26.6	20	66.7	2	6.7
Total	80	15		57		8	
Mean, %			18.7		71.3		10.0
